# Collagen treatment of complex anorectal fistula: 3 years follow-up

**DOI:** 10.1515/med-2022-0553

**Published:** 2023-07-11

**Authors:** Matteo Maternini, Angelo Guttadauro, Pasquale Avella, Antonio Buondonno, Domenico Mascagni, Giovanni Milito, Angelo Stuto, Adolfo Renzi, Maria Rennis, Corrado Bottini, Gennaro Quarto, Raffaele Nudo, Luca Del Re, Bruno Amato, Francesco Gabrielli

**Affiliations:** General Surgery Department, Istituti clinici Zucchi of Monza, University of Milano-Bicocca, 20126, Milan, Italy; Department of Medicine and Health Sciences “V. Tiberio,” University of Molise, Via Francesco De Sanctis 1, 86100, Campobasso, Italy; General Surgery Department, Policlinico Umberto I, University La Sapienza of Rome, 00161, Rome, Italy; General Surgery Department, University Hospital of Roma “Tor Vergata,” 00133, Rome, Italy; General Surgery Department, IRCCS Policlinico San Donato of Milano, 20097, Milan, Italy; General Surgery Department, Clinica Villa Delle Querce, 80136, Naples, Italy; General Surgery Department, Ospedale San Gerardo di Monza, University of Milano-Bicocca, 20900, Milan, Italy; General Surgery Department, Hospital of Gallarate, 21013, Varese, Italy; Department of Clinical Medicine and Surgery, University of Naples “Federico II,” Via S. Pansini, 5, 80131 Naples, Italy; General Surgery Department, Casa di Cura Fabia Mater, 00171, Rome, Italy; General Surgery Department, Ospedale Multimedica San Giuseppe of Milano, 20123, Milan Italy; Department of Public Health, University of Naples “Federico II,” Via S. Pansini, 5, 80131 Naples, Italy

**Keywords:** complex anal fistula, Salvecoll, Permacol, sphincter-sparing treatment, non-cutting technique, mini-invasive treatment

## Abstract

Fistula in ano is a common anorectal disease in adults. Currently, surgery remains the definitive therapeutic approach, but in some cases, it can lead to serious complications as faecal or gas incontinence. Therefore, sphincter sparing treatments should be considered for complex fistulas. One of the sphincteric preserving treatment is the filling with a dermal extract commonly called “collagen glue” as Salvecoll-E^®^ gel. This is a multicentric, prospective, observational study on the use of Salvecoll-E^®^ gel in treatment of complex anal fistulas. We treated 70 patients from May 2016 to May 2017. In the first phase, we debrided the fistula tract using a loose seton kept for 4–6 weeks. In the second phase, the seton was removed and the fistula tract was filled with Salvecoll-E^®^ gel. In this article, we report results at 36 months of follow-up. Fifty patients (71.4%) had completely healed fistula within 36 months of follow-up. Twenty-eight patients (28.2%) had recurrences. Among these failures, 65% were within 6 months. All low transphincteric fistulas healed. Recurrences occurred only in median and high transphincteric fistulas. No patient had a worsening of continence status measured with Cleveland Clinic Florida Incontinence Severity score. Salvecoll-E^®^ gel is a recent finding among sphincter-sparing treatments. In this study, we demonstrate that it is a safe option in the treatment of complex fistulas. Final results are satisfactory and in line with the best results published in literature among mini-invasive treatments.

## 1 Introduction

Fistula in ano is a tract that connects the anal canal to the perineal skin. A fistula develops ex novo or in 40–60% of cases it is the result of the drainage of an abscess produced by the obstruction of a gland [1].

It is a common anorectal disease in adults with an incidence of 8.6–10 per 100,000 people per year that varies among different countries in Europe [2]. It is more frequent in male patients with a ratio of 2:1 [3].

Parks classified 5 types of fistulas based on anatomical position in relation with the external and internal sphincter [4].

A fistula can also be categorised as simple or complex. “Complex” anal fistulas are suprasphincteric, extrasphincteric, transphincteric fistulas that involve more than 30% of the external sphincter, recurrent fistulas, and anal fistulas associated with inflammatory bowel disease, radiation, malignancy, or diarrhoea [5].

To diagnose fistulas, an imaging may be necessary in addition to clinical examination; pelvic MRI is one of the most used technique due to high sensitivity (90%) [6].

While the surgical environment has changed dramatically over the past 20 years with the introduction of new technologies and mini-invasive surgical approaches that improve short-term outcomes in several surgical areas [7,8,9,10,11], the anorectal fistulas treatment is still debated [12].

Since the past, surgery was the principal treatment of anal fistulas through fistulotomy or the seton use [13] and it remains the definitive therapeutic approach. Fistulotomy is the first surgical approach for simple anal fistulas, it is a low-cost treatment and reaches a healing score of more than 90% of the patients [14,15], but it can be a challenge for the surgeon in case of a complex fistula involving sphincters [12,16]. Furthermore, surgical treatment can lead to serious complications including faecal and/or gas incontinence in more than 40% of patients [17] and soiling when performed for complex fistulas [18]. Therefore, sphincter-sparing treatment should be considered for complex fistulas [12]; one of the these treatments is filling the fistula tract with a biological glue. As we have described in a previous article [19], one of the biological glues that can be used is Salvecoll-E gel, an equine collagen like the human one obtained from the derma of equines, which is purified and deprived from the antigen. Structurally it is a non-cross-linked collagen, so it is easily degradable and more tolerable for human body.

When Salvecoll-E^®^ gel is injected into the fistula tract, first, it refills mechanically the defect. In the second time, it realises an acellular matrix which stimulates the immune system where inflammatory cells as fibroblasts and macrophages migrate these cells gradually produce AN inflammatory response that destroys equine collagen and produce human collagen leading to a natural healing of the fistula tract. This article is a conclusion of an observational, prospective study published in 2019 [19].

In the previous prospective, observational, multicentric clinical study we have shown the healing rate, the recurrences, and the preservation of continence in patients with anal fistula treated with Salvecoll-E^®^ gel after 12 months of follow up [19]. In this second article, we analyse the recurrence rate after 36 months of follow up using the same sample of patients to conclude our discussion.

## 2 Materials and methods

This is a multicentric, prospective, single-arm observational study on the use of Salvecoll-E^®^ gel in the treatment of complex anal fistulas. We analysed 70 patients of 9 Italian centres. This study was conducted according to Good Clinical Practice guidelines and was approved by local and regional Ethics Committees, it follows all European and National regulations.

Between May 2016 and May 2017, 70 patients with “complex” anal fistula were treated with 150 mg/ml of Salvecoll-E^®^ gel. To define a fistula “complex” we used the American Society of Colon and Rectal Surgeons definition [20]. Inclusion and exclusion criteria are summarised in Table 1.

**Table 1:** Inclusion and exclusion criteria

Inclusion criteria	Exclusion criteria
Age >18 years Anal fistulas of glandular cryptic origin Complex fistulas Presence of signed informed consent	Chronic inflammatory bowel diseases Abscess and acute anal sepsis in progress Immunosuppressive therapy Rectovaginal fistulas Infectious diseases, HIV, tuberculosis, hidradenitis suppurativa, pilonidal sinus disease Radiation treatment of the pelvic region Anal and/or rectal carcinoma Previous incontinence with a score >2 according to Wexner Continence Grading Scale Anorectal diseases of infectious, actinic endocrine, or drug origin Patients with clinically significative hepatic, renal, haematological, cardiovascular, pulmonary, neurological, psychiatric, immunological, gastrointestinal, endocrine disease Lactating or pregnant women Malignant neoplasia of any kind or history of previous malignancy Abuse of alcohol, drugs, or psychotropic medicaments that can change mental status in terms of vigilation and perception of reality Presence of dementia or mental deterioration that can invalidate the ability to take prescribed therapy or to attend the follow-up Other conditions that can determinate absence of compliance to the study protocol Previous participation in this study

Before treatment, patients underwent clinical examination and pelvic MRI [21]. Colonoscopy was reserved only to patients with risk factors of colon-rectal cancer.

Each patient filled a questionnaire to establish faecal continence according to Wexner/Cleveland Clinic Florida Faecal Incontinence (CCF-FI) score and to stratify the gravity of symptoms [22]. An extensive technical notes report was described in our previous experience [19]. Post-operative pain was measured with visual analogic scale [23].

Follow-up was done after 7 days and after 1, 3, 6, 12, 24, and 36 months after the surgery, through telephone interview and regular outpatient visits [24].

The primary endpoint, that is the healing rate, was defined as the absence of material draining from fistula’s tract and the closure of the external orifice.

Secondary endpoint, the faecal/gas continence, was calculated using Wexner/CCF-FI score [22]. We published the first results at 1 year of follow-up [19]. In this article, we publish the data collection after 3 years of follow-up.

## 3 Results

No patient was lost in follow-up before the 36 months visit. Demographic characters and types of fistulas are shown in Table 2. Mean time between the insertion of the seton and the injection of Salvecoll-E^®^ gel was 36 days. After removal of the seton, all fistulas were debrided from granulation tissue. A single syringe of Salvecoll-E^®^ gel (150 mg/ml) was enough to fill fistula’s tract in all patients. No patient had any adverse reaction after the procedure.

**Table 2:** Demographics and fistula characteristics

Mean age, years (range)	45.6 (25–78)
**Sex,** ***n*** **(%)**	
Female	18 (26)
Male	52 (74)
Mean body mass index, (range)	27 (24–38)
Mean ASA grade	2
**Type of fistula,** ***n*** **(%)**	
Low transphincteric	17 (24.3)
Median transphincteric	35 (50)
High transphincteric	18 (25.7)
Prior treatment with loosing seton, *n* (%)	70 (100)
Mean time between placement and removal of seton, days (range)	36 (28–43)
Average length of hospital stay, days	1

The average duration of the procedure was 24 min (range: 10–45 min). All patients were discharged within 24 h and returned to their usual daily life after approximately 6 days (range: 1–10 days). Fifty patients (71.4%) had completely healed fistula within 36 months of follow up. Twenty patients (28.6%) had recurrences.

**Figure 1:**
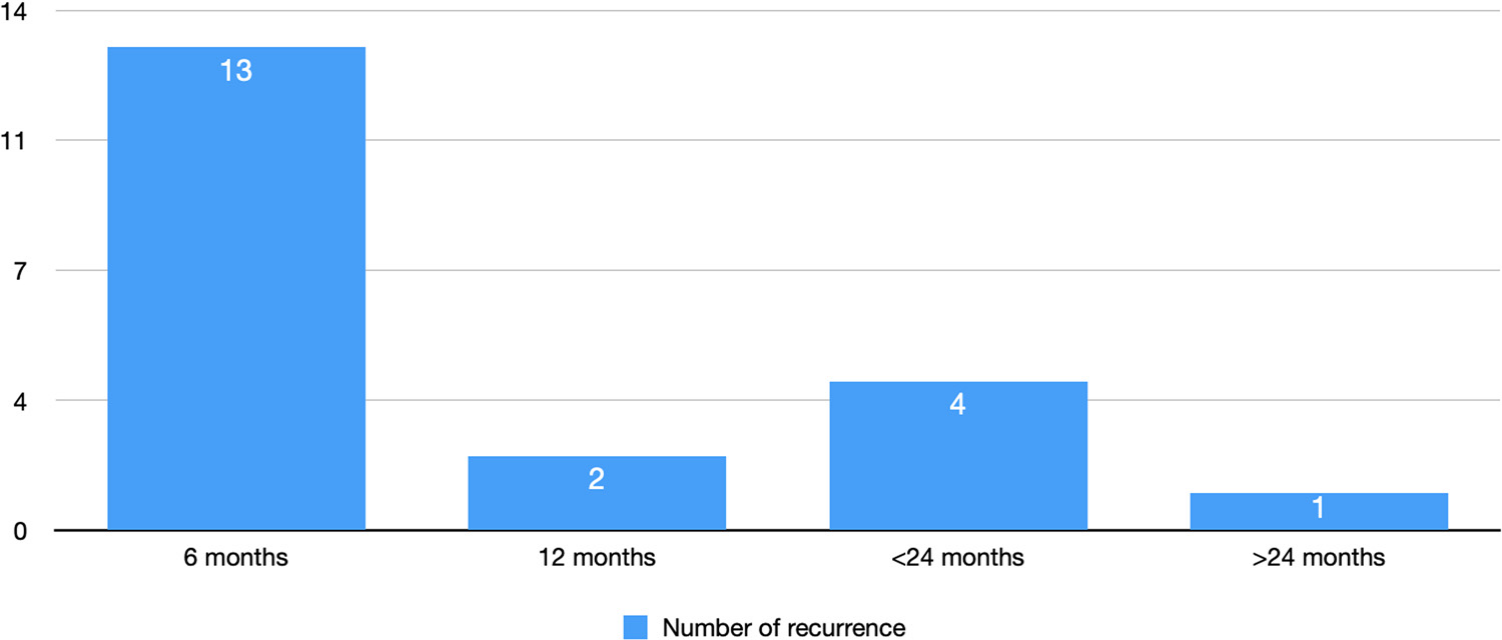
Rate of recurrence at 6, 12, 24, and >24 months of follow-up.

After 1 year of follow-up, recurrences occurred in 15 patients (25%). Majority of the recurrences were registered in the first 6 months (65%) (Figure 1). All low transphincteric fistulas healed.

Recurrences occurred in high and median transphincteric fistulas in 100% of cases. No patient had a worsening of his continence status measured with CCF-IS. Patients were asked about their overall satisfaction after the last follow up with ratings from very satisfied to very unsatisfied. Fifty-one patients (72.8%) were either satisfied or very satisfied. Results are summarised in Table 3 and in Figure 1.

**Table 3:** Summary of recurrences using Salvecoll-E® gel technique

Mean Age, years	42
Number of recurrences, *n* (%)	20 (28.6)
**Period of recurrences,** ***n*** **(%)**	
<6 months	13 (65)
<12 months	2 (10)
<24 months	4 (20)
>24 months	1 (5)
**Type of Fistula,** ***n*** **(%)**	
Low transphincteric	0 (0)
Median transphincteric	11 (55)
High transphincteric	9 (45)

## 4 Discussion

In our experience, Salvecoll-E gel is a good treatment for anal fistulas with a rate of resolution around 70%. It is a safe and non-invasive treatment that could be used even in frail patients. Recurrences after this treatment is relatively low (around 30%) especially for high transphincteric fistulas.

Lately there have been new surgical approaches used to treat complex anal fistulas with the aim to preserve anal continence [12]. Examples of sphincter-preserving methods are fistula laser closure, video-assisted anal fistula treatment, ligation of the intersphincteric fistula tract, over-the-scope clip, and filling with fibrin glue or with stem cells matrix [25,26,27,28,29].

The use of animal collagen products is a recent discovery in surgery, if considered the application of synthetic monomers in numerous fields [19,30,31,32,33,34]. In previous studies, a product like Salvecoll-E^®^ gel used to tract complex fistulas was Permacol™ collagen paste (Covidien plc, Gosport, Hampshire, UK) [35]. Permacol is a porcine dermal collagen matrix that, unlike Salvecoll, has a cross-linked structure [36]. The success rate of Permacol™ reaches 50% at 2 years according to literature [37,38,39].

Our results seem to be in line with the best results published in literature until now [38,40].

Analysing results, we could suppose that one of the risk factors of recurrence is the type of fistula. High and median transphincteric fistula recurrences more than low ones.

For this reason, in the group of complex fistulas, we could propose a new definition that the low transphincteric fistulas are “less” complex than the high and median transphincteric ones. Another interesting finding is that most of the recurrences happen in the first 6 months. This helps to understand which patients could benefit from other treatments in less than 6 months.

A strength of this procedure is the reversibility in case of recurrence; it is a non-invasive and non-demolitive technique that does not exclude the possibility to use other approaches in case of failure.

Another advantage is that it could be done in day-surgery, and it is inexpensive in terms of length of stay in hospital.

A point in favour of our study is the long follow-up that consolidates results after the first year of follow-up. Another encouraging characteristic of Salvecoll-E^®^ gel is his tolerability. In an inflammatory condition, as the fistula, the presence of many cells of the immune system can promote a rejection easier than in a healthy condition. Therefore, using a material more degradable as a non-cross-linked collagen (Salvecoll) can be more tolerable.

### 4.1 Limitations

This study has obviously some weaknesses, the number of patients is low, and it would be useful to recruit a large number of participants to strengthen the results. Moreover, our study is not a randomised control trial, there is no control group to compare our findings with other treatments.

Moreover, another bias is represented by the exclusion of patients affected by horseshoes fistulas. These types of fistulas usually are associated with perineal sepsis and need invasive treatments with extended resections [41].

## 5 Conclusion

Salvecoll-E^®^ gel could be an effective and safe technique in the complex treatment of anorectal fistulas, taking into account the long-term outcomes. Unfortunately, it is difficult to compare different sphincter-sparing techniques based on literature because there may be demographic differences between the patients involved and differences among the fistula types.

To obtain stronger evidence, other studies are needed, involving a larger number of patients. Therefore, a clinical trial should be done to strengthen the evidence of our study.
